# Targetedly Attenuating Cancer Stemness and Plasticity by Homologous Cancer Stem Cell‐Inherited Fusion Membrane Nanoeffectors against Cancer Metastasis

**DOI:** 10.1002/smsc.202300111

**Published:** 2023-12-03

**Authors:** Xiulin Dong, Qiaoling Yang, Hai Wang, Chunyan Zhu, Taixia Wang, Chao Fang, Yan Zhang, Jianjun Yang, Kun Zhang, Qing Zhao

**Affiliations:** ^1^ Department of Pharmacy and Central Laboratory Sichuan Academy of Medical Sciences Sichuan Provincial People's Hospital School of Medicine University of Electronic Science and Technology of China No. 32, West Second Section, First Ring Road Chengdu 610072 Sichuan P. R. China; ^2^ Department of Orthopaedics and Department of Medical Ultrasound Shanghai Tenth People's Hospital Tongji University School of Medicine Tongji University No. 301 Yan-chang-zhong Road Shanghai 200072 P. R. China; ^3^ In-Patient Ultrasound Department Second Affiliated Hospital of Harbin Medical University Surgeons’ Hall, No.246. XuefuRoad, Nangang District Harbin Heilongjiang China

**Keywords:** cancer stem cells, cancer stemness and plasticity attenuation, energy metabolism disruption, fusion membranes, tumor metastasis and recurrence

## Abstract

Cancer stem cells (CSCs) are highly related to initiate metastasis and drive tumor relapse/propagation, and the induced cancer stemness and plasticity render cancer intractable to various therapeutic approaches. To address them, a CSC membrane‐based fusion strategy is presented to target and attenuate cancer stemness and plasticity, and accordingly homogenous CSC‐inherited fusion membrane‐coated biomimetic nanoeffectors based on doxorubicin‐loaded hydroxyapatite nanoparticles are engineered. Such biomimetic nanoeffectors coated with fusion membrane consisting of CSCs and cancer cells can not only specifically target the solid breast tumor cells, but also target the latent CSCs. Systematic experiments validate that the constructed nanoeffectors disrupt energy metabolism, trigger lethal mitochondrial apoptosis, remarkably inhibit tumor proliferation, and downregulate the expressions of some target proteins associated with cancer stemness and metastasis to attenuate cancer stemness and the induced plasticity. Contributed by them, such nanoeffectors have been demonstrated to successfully hinder the progression of breast cancer, and decrease lung metastasis in two cancer metastasis mouse models. Such CSCs membrane‐based fusion membrane strategy provides a new candidate avenue rather than the currently dominant immune‐related pathway to repress cancer metastasis via targeting and attenuating cancer stemness.

## Graphical abstract

A homogenous cancer cell (CC) and cancer stem cell (CSC) membrane fusion strategy and the corresponding CCs–CSCs fusion membrane‐coated biomimetic nanoeffectors based on doxorubicin‐loaded hydroxyapatite nanoparticles have been designed to target both CCs and CSCs, attenuate, and even reverse the cancer stemness and plasticity of breast cancer, which effectively prevents cancer progression and minimizes their recurrence and lung metastasis.

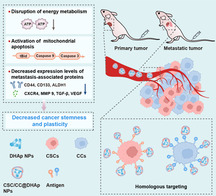

## 1 Introduction

The unremitting propagation and metastasis of malignant tumors pose a significant challenge to clinical cancer therapy.^[^ ^1^, ^2^ ^]^ The main strategies to inhibit such metastasis of malignant tumors highlight the use of matrix metalloproteinase inhibitors, angiogenesis inhibitors, and immunotherapeutic agents.^[^ ^3^, ^4^ ^]^ Typically, a pH responsive gel that entrapped calcium‐containing inorganic salts and an immunoadjuvant R837 were reported to prevent tumor recurrence and metastasis by triggering systemic immune responses.^[^ ^5^ ^]^ However, these researches fail to eradicate and wipe out the seed and soil that favor cancer metastasis at the source, e.g., cancer stem cells (CSCs) and its complex microenvironments. CSCs as a subpopulation of tumor cells with the capacity of self‐renewal and differentiation have been identified as the seeds to initiate propagation and aggravate cancer progression,^[^ ^6^, ^7^, ^8^, ^9^ ^]^ which is recognized as another dominant pathogenesis of tumor metastasis and disable postoperative recovery in cancer patients.^[^ ^10^ ^]^ The presence of CSCs can reinforce the cancer stemness and plasticity, and render cancer intractable against various treatment methods. Given that, the chase for effective strategies that target CSCs and reverse cancer stemness and attenuate cancer plasticity has become as a crucial concern for clinical cancer therapy. However, the nonspecificity, off‐target effect and inadequate internalization within CSCs render cancer stemness targeting intractable.^[^ ^11^, ^12^ ^]^

Recent advances in cancer biology reveal that cancer cells (CCs) whose membrane possesses specific antigen expression profiles which are crucially involved in the homologous cell adhesion within tumors microenvironment.^[^ ^13^, ^14^ ^]^ By coating extracted CC membrane on nanoparticles (NPs), the resulting biomimetic NPs can retain the antigenic structure and function from their parent cells.^[^ ^15^ ^]^ Based on this, a series of CC membrane‐camouflaged NPs were developed and showed desirable features inherited from source cells, e.g., targeted drug delivery and adequate antigen supply.^[^ ^16^, ^17^, ^18^ ^]^ Especially after fusion with dendritic cells (DCs), the DCs–CC membrane fusion enabled more robust immune activation.^[^ ^19^ ^]^ However, current cell membrane encapsulation fails to target CSCs and the absence of effectively attenuating cancer stemness remains an obstacle to repress the metastasis of malignancies such as breast cancer with high metastatic rate.^[^ ^1^, ^20^, ^21^ ^]^

**Figure 1:**
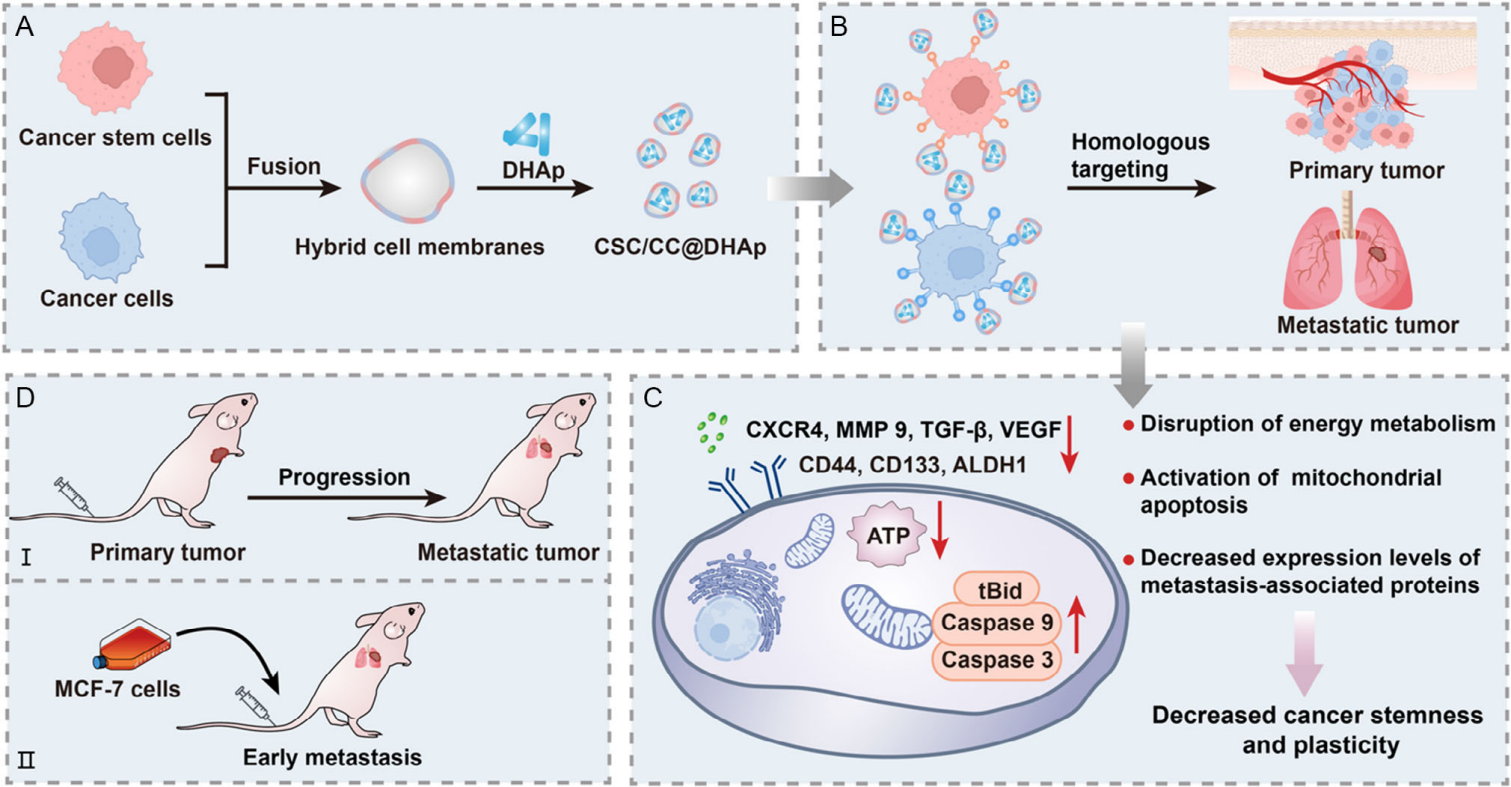
Schematic illustration of biomimetic fusion membrane‐coated nanoeffector (CSC/CC@DHAp NPs) for hindering tumor progression by attenuating the stemness and plasticity of breast cancer. A,B) Herein, CSCs and CCs fusion membrane were coated onto DHAp to obtain CSC/CC@DHAp, and obtained nanoeffectors can realize the homologous targeting of CCs and CSCs in primary and metastatic tumor. C) Afterward, they can disrupt energy metabolism, activate mitochondrial apoptosis (upregulations of tBid, Caspase 9 and Caspase 3), decrease metastasis and stemness‐associated proteins expression (e.g., CXCR4, MMP9, TGF‐β, VEGF, CD44, CD133, and ALDH1), and eventually attenuate the stemness and plasticity of breast cancer, D) which not only inhibit the progression and metastasis of primary tumor, but also repress early metastasis of breast cancer.

In this report, a homogenous CC and CSC membrane fusion strategy for targeting and attenuating or even reversing the cancer stemness and plasticity has been designed. Accordingly, doxorubicin (DOX)‐loaded hydroxyapatite NPs (DHAp NPs) coated with hybrid membranes consisting of CCs–CSCs membrane fusion were engineered to obtain the biomimetic nanoeffectors (CSC/CC@DHAp NPs) for realizing the targeted attenuation of cancer stemness and the induced plasticity to improve antitumor and antimetastasis efficiencies (**Figure** **1**). Depending on the homologous tropism, the coated CCs–CSCs membrane fusion system is expected to not only specifically target the solid tumor cells, but also target the elusive CSCs that reside within the body,^[^ ^22^ ^]^ thus enabling the attenuations of cancer stemness and plasticity (Figure 1). Our previous work unveiled that DHAp NPs could effectively suppress multidrug‐resistant breast cancer,^[^ ^23^ ^]^ and DOX has been documented to trigger strong toxic effects, including DNA damage and cell membrane disruption.^[^ ^24^ ^]^ Regarding this, the resulting NPs may target and kill the intrinsic metastasis seed (CSCs), effectively prevent tumor progression, coincidently minimize the risk of recurrence, and inhibit tumor metastasis initiated by CSCs (Figure 1), all of which have not been involved in previous drug‐loaded HAp nanosystems. Contributed by them, the constructed biomimetic nanoeffectors successfully targeted breast cancer and CSCs, disrupted energy metabolism, and activated lethal mitochondrial apoptosis. Moreover, they successfully downregulated the expressions of CD44, CD133, and ALDH1 proteins that correlate with cancer stemness, and inhibited the secretions of metastasis makers including CXCR4, VEGF, TGF‐β, and MMP 9, which contributed to the attenuated cancer stemness and plasticity and the significantly inhibited progression and lung metastasis of breast cancer in situ.

Currently, immune activation or potentiation or combination with immunotherapy have been highlighted and attracted increasing interests,^[^ ^25^, ^26^, ^27^, ^28^, ^29^ ^]^ which have been identified as the main stream pathway to suppress cancer metastasis. Herein, completely differing from them, the innovative CSCs membrane‐based fusion membrane strategy and this application in attenuating stemness and plasticity of malignant cancer that have not been reported yet will provide a new but reliable option to potentiate antitumor and antimetastasis efficiency of malignancies.

## 2 Results and Discussion

### 2.1 Biomimetic CCs–CSCs Hybrid Membrane‐Coated Nanoeffectors (CSC/CC@DHAp NPs) Engineering

**Figure 2:**
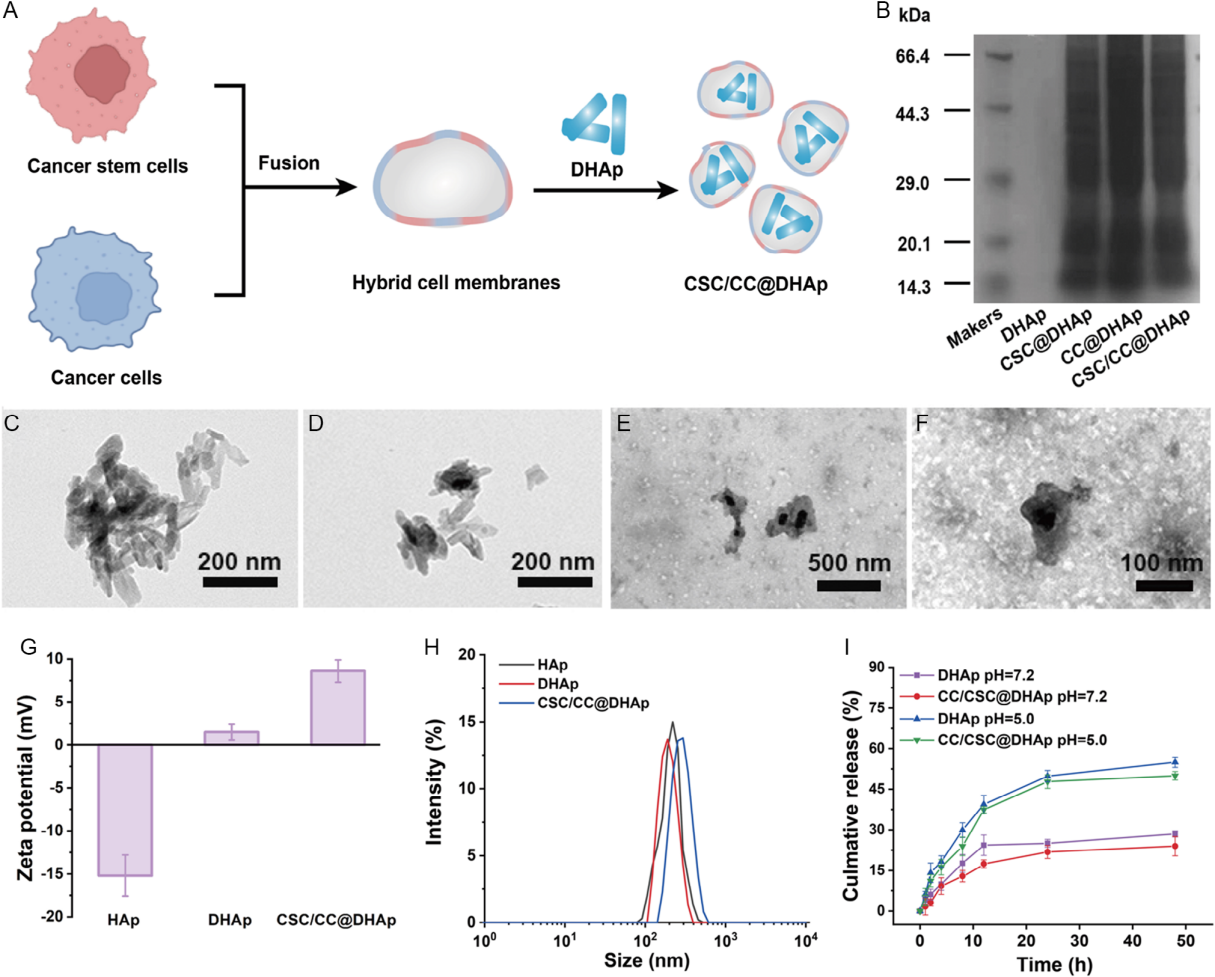
Synthesis and characterization of CSC/CC@DHAp NPs. A) Diagram of the preparation of fused membrane‐coated DHAp NPs. B) SDS–PAGE analysis of different samples. C–F) TEM images of C) HAp, D) DHAp, E,F) CSC/CC@DHAp NPs. G,H) Zeta potential (G) and hydrodynamic size distribution (H) of HAp, DHAp, and CSC/CC@DHAp. I) Cumulative release of DOX from DHAp, and CSC/CC@DHAp at different pH values (7.4 and 5.0, corresponding to that in blood and endosomes/lysosomes, respectively) in 48 h. Error bars show the standard deviation (*n* = 3).

CSCs promote the relapse or diaspora of primary tumor, which thereby determines that attenuating or even reversing tumor stemness can serve as a general and promising strategy to inhibit tumor metastasis. With targeting the plasticity induced by cancer stemness, we developed a biomimetic nanoeffector coated with CCs–CSCs hybrid membrane, which could target both parent CCs and CSCs for inhibiting tumor growth and recurrence. Herein, HAp NPs were used as carriers because it features high biocompatibility and specific antitumor activity via disrupting intratumoral Ca^2+^ homeostasis.^[^ ^23^ ^]^ The fusion membrane‐coated NPs, i.e., CSC/CC@DHAp, are constructed, as illustrated in **Figure** **2**A. In detail, to obtain hybrid membrane, we first collected CSCs and CCs membranes from their parent cells (MCF‐7). CSCs were enriched via a sphere formation assay which served as a well‐established method for enriching stem cells according to previously described articles,^[^ ^30^, ^31^ ^]^ followed by the removal of intracellular contents to obtain CSC membranes. The two acquired cell membranes were then mixed by sonication to obtain CCs–CSCs membrane fusion system. Finally, DHAp NPs were coated with the hybrid fusion membranes to acquire the resulting CSC/CC@DHAp NPs. The encapsulation efficiency and loading percentage of DOX in the nanaoeffectors are determined to be 65% and 9%, respectively. Successful coating was confirmed by sodium dodecyl sulfate–polyacrylamide gel electrophoresis (SDS–PAGE) analysis and TEM images. As shown in Figure 2B, characteristic protein bands are clearly observed in CSC/CC@DHAp, whereas DHAp alone exhibits no discernible protein bands. TEM images show the presence of visible core–shell structure in the CSC/CC@DHAp NPs, which is not found in the uncoated NPs (Figure 2C–E). These results indicate the successful coating of DHAp NPs by CCs–CSCs fusion membrane. The hydrodynamic diameter of hybrid membranes NPs is slightly increased and the zeta potential rises from 1.5 to 8.6 mV after coating with the fused cell membranes, further demonstrating the successful preparation of CSC/CC@DHAp NPs (Figure 2F,G). The release profiles of DOX from DHAp and CSC/CC@DHAp NPs exhibited a typical sustained and pH‐dependent release (Figure 2H). In detail, an initial burst drug release from DHAp and CSC/CC@DHAp NPs appears in the first 12 h, and then the release level reaches a plateau as the incubation time at different pH values is prolonged. The accumulative release percentage of DOX from CSC/CC@DHAp NPs reaches to 28.5% at pH = 7.4. In contrast, the release rate exceeds 50% at pH = 5.0. Such a pH‐responsive release enables the precise and specific nanomedicine against tumor, which will promote the therapeutic efficiency. Meanwhile, cell membrane coating slightly delays the drug release rate compared to uncoated DHAp NPs, hopefully realizing the continuous drug release and the sustained action.

### 2.2 CCs and CSC Targeting Test Based on the CCs–CSCs Fusion Membrane‐Coated Nanoeffectors

**Figure 3:**
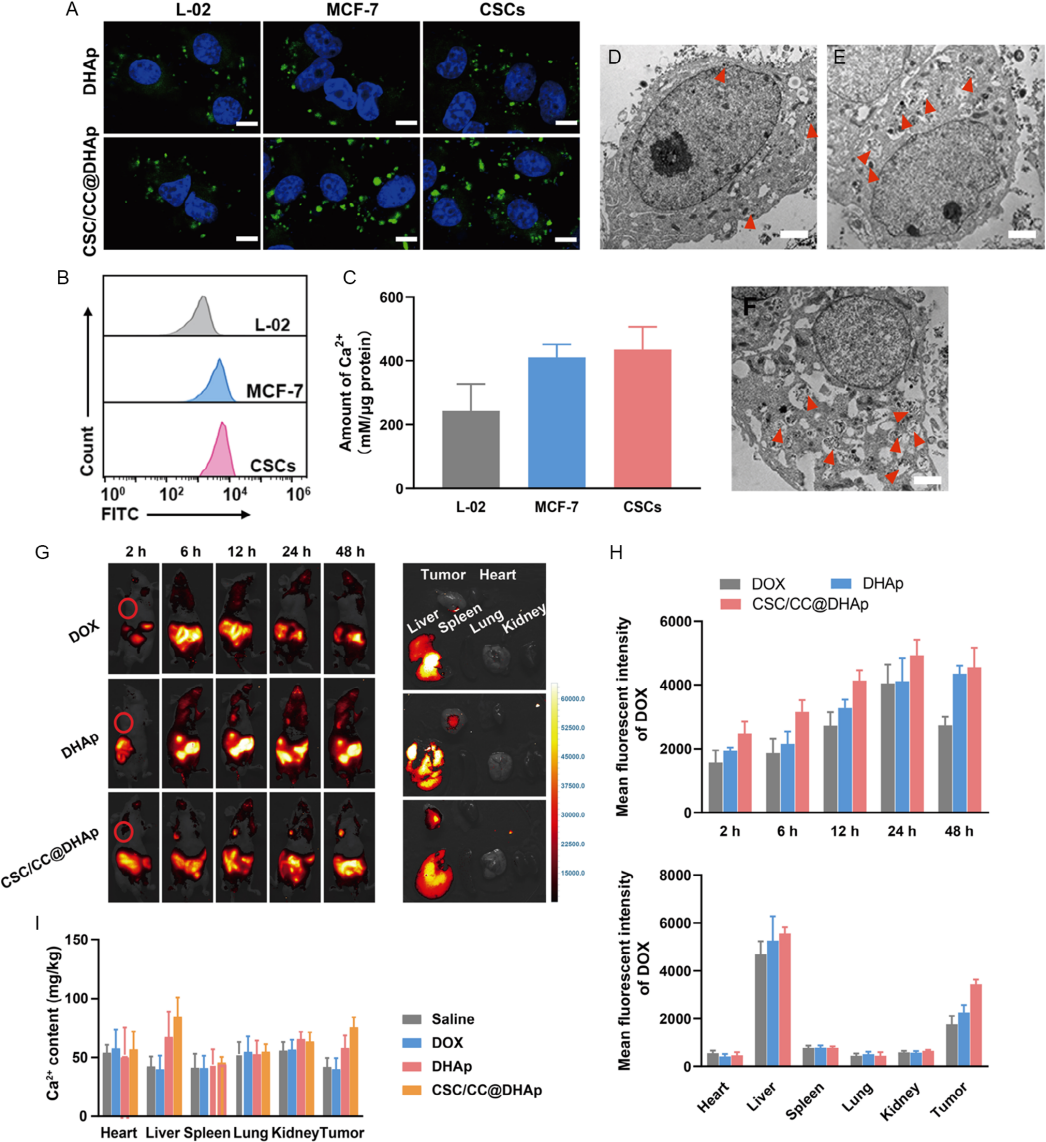
CSC‐targeting property of CSC/CC@DHAp nanoeffectors. A) Cellular uptake of FITC‐HAPNs by L‐02, MCF‐7, and CSCs pretreated with DHAp, CSC/CC@DHAp for 4 h, respectively, and the excitation/emission wavelengths of FITC and DOX are 488 nm/520 nm and 488 nm/630 nm, respectively; and scale bars: 20 μm. B) Cellular uptake of FITC‐labeled CSC/CC@DHAp via FCM analysis. C) Amount of Ca^2+^ intake detected by ICP‐OES. Error bars showed the standard deviation (*n* = 3); *P < 0.05, **P < 0.01, and ***P < 0.001. D) Representative bio‐TEM images of L‐02, E) MCF‐7, and F) CSCs after CSC/CC@DHAp treatments for 4 h, wherein CSC/CC@DHAp NPs were marked with orange arrows. Scale bars: 4 μm. G) In vivo biodistribution of DOX, DHAP, and CSC/CC@DHAp NPs in MCF‐7 tumor‐bearing nude mice at different time points after i.v. injection. Red circles indicate the tumors. (Dose: 5 mg kg^−1^); Ex vivo biodistribution of DOX, DHAP, and CSC/CC@DHAp NPs in tumor and major organs (heart, liver, spleen, lung, and kidney) after 48 h injection. H) Quantification of fluorescence intensity of DOX from the fluorescent images in (G). I) Biodistribution of CSC/CC@DHAp NPs in mice after 48 h injection determined by ICP‐OES‐measured Ca^2+^ concentrations.

After successfully confirming fusion membrane coating on DHAp, we investigated the homologous targeting capability of biomimetic NPs both in vitro and in vivo. First, normal liver cells (L‐02), breast CCs (MCF‐7), and the corresponding CSCs were incubated with FITC‐labeled CSC/CC@DHAp NPs for 4 h, and their targeting capability was surveyed by confocal laser scanning microscopy and flow cytometry (FCM), respectively. Bright green fluorescence with similar intensity is observed in MCF‐7 and CSCs after incubation with CSC/CC@DHAp NPs, while the fluorescence in normal cells is weak (**Figure** **3**A). Specifically, the fluorescent intensity of MCF‐7 and CSCs after incubation with fusion membrane‐coated NPs is stronger than that of DHAp NPs, where the relative fluorescent intensity is elevated from 2.2 to 4.2 in CSCs and 2.2 to 4.0 in MCF‐7 cells (Figure 3A and S1, Supporting Information), respectively. These convincing data reliably verify the specific targeting ability of CCs–CSCs fusion membrane toward tumor cells and the corresponding CSCs. Similar results are found in FCM assay (Figure 3B and S2, Supporting Information), wherein more fusion membrane‐coated nanoeffectors accumulate in CSCs. Additionally, these cells containing CSC/CC@DHAp NPs after coculture were collected to further detect the targeting ability of NPs by inductively coupled plasma optical emission spectrometer (ICP‐OES) and bio‐TEM. It is found that the total amount of Ca^2+^ in CSCs or MCF‐7 cells is greatly increased by almost 0.7‐fold compared to that in L‐02 cells (Figure 3C). Meanwhile, bio‐TEM image reveals an increased abundance of NPs marked with orange arrows in CSCs or MCF‐7 cells as compared to the normal cells (Figure 3D). This disparity in cellular internalization is ascribed to the homologous targeting ability originated from the source cells.

Subsequently, to investigate the specific tumor‐targeting ability of CSC/CC@DHAp NPs in vivo, MCF‐7 tumor‐bearing nude mice were intravenously injected with free DOX, DHAp, and CSC/CC@DHAp NPs, and then in vivo imaging was performed at different time points. The fluorescence signals at the site of tumor show a gradual increase after injections of DHAp and CSC/CC@DHAp NPs, whereas the fluorescence signal of free DOX displays a more widespread distribution throughout the body with a rapidly decreased trend after 24 h (Figure 3G). Noticeably, the mice treated with CSC/CC@DHAp NPs show the highest fluorescence signal at the site of tumor, reflecting the excellent cancer‐targeting capability of fusion membrane‐coated NPs in vivo. Afterward, all mice were euthanized and their major organs and tumor tissues were harvested for further analysis. Ex vivo fluorescence images show a relatively higher drug accumulation and retention in the tumor and liver compared to other organs. Inspiringly, the tumor tissues collected from CSC/CC@DHAp NP‐treated mice show the highest fluorescence intensity compared to DOX or DHAp treatment (Figure 3H), suggesting the most accumulation, which is consistent with the results determined by ICP‐OES (Figure 3I). These data support that CSC/CC@DHAp NPs possess a favorable and specific CCs and CSC‐targeting property both in vitro and in vivo, which pave a solid foundation to inhibit tumor growth, reverse CSC‐arised cancer stemness, and hinder CSC‐mediated tumor progression and metastasis.

### 2.3 Biological Activity Disruption and Stemness Attenuation of Breast CSCs via Mitochondrial Apoptotic Pathway

**Figure 4:**
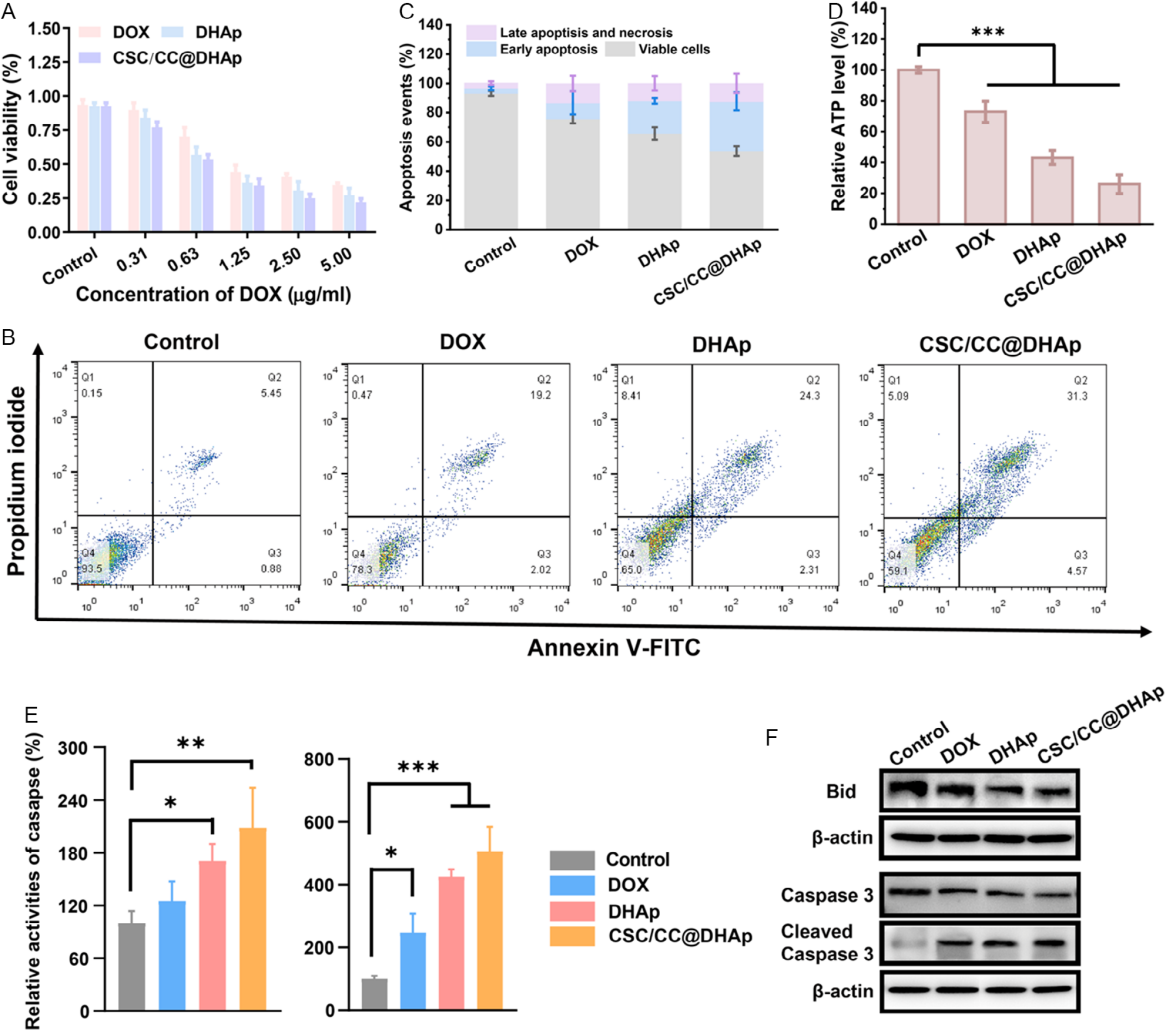
Perturbation of biological activity of breast CSCs by CSC/CC@DHAp nanoeffectors. A) Cell viability of CSCs exposed to free DOX, DHAp and CSC/CC@DHAp NPs, respectively, at various concentrations for 24 h (*n* = 4). B) Cell apoptosis analysis of CSCs assessed with FCM following Annexin V‐FITC/PI double staining. C) Corresponding apoptosis ratios quantified from Figure B. D) Intracellular ATP level of CSCs after incubation with free DOX, DHAp, and CSC/CC@DHAp NPs for 24 h. The relative ATP level and fluorescence intensity was expressed as the percentage of that in the control cells without any treatment. Error bars showed the standard deviation (*n* = 3); *P < 0.05, **P < 0.01, and ***P < 0.001. E) Activity of caspase‐9 (left) and caspase‐3 (right) in CSCs (*n* = 3). Cells without any treatment were used as the control to calculate the relative caspase activity. *P < 0.05, **P < 0.01, and ***P < 0.001. F) WB assays of protein caspase‐3, cleaved caspase‐3, and Bcl‐2 in CSCs after different treatments for 24 h.

Encouraged by the excellent CSC‐targeting property of CSC/CC@DHAp NPs in vitro and in vivo, we tested the cytotoxic effects and stemness attenuation of NPs on CSCs. The cell counting kit‐8 assay reveals that free DOX, DHAp, and CSC/CC@DHAp NPs all exhibit a dose‐dependent toxicity to CSCs. Notably, the viability of CSCs after treatment with CSC/CC@DHAp NPs is obviously lower than that treated with DOX or DHAp NPs at equivalent DOX concentration, which might be ascribed to the CSC‐targeting‐encouraged accumulation of CSC/CC@DHAp NPs. The results were also supported by Annexin V‐FITC/PI double staining assay. After incubation with free DOX for 24 h, the apoptosis and necrosis percentages of CSCs are only 24.1%, but it reaches 46.2% for CSC/CC@DHAp NPs treatment (**Figure** **4**B,C).

Subsequently mechanistic analysis reveals that mitochondrial apoptotic pathway in CSCs treated with CSC/CC@DHAp NPs is found to be activated. ATP production is inhibited in CSCs after exposure to CSC/CC@DHAp NPs (Figure 4D), which is reduced to 26.0% compared to untreated cells. Furthermore, CSC/CC@DHAp NPs treatment activates caspase‐9 (mitochondrial apoptotic marker) and caspase‐3 (apoptotic executor) with 1.1‐ and 4.1‐folds higher activity than that in the control group (Figure 4E), respectively. Western blot (WB) assays also show the upregulation of cleaved caspase‐3 and the downregulation of caspase‐3 in the presence of CSC/CC@DHAp NPs (Figure 4F). Concurrently, the expression level of full‐length Bid is decreased to 1/2 level of that in untreated CSCs (Figure 4F and S3, Supporting Information), implying that Bid is cleaved to truncated form of Bid (tBid), leading to mitochondria‐dependent apoptosis. Similar to CSCs, mitochondrial apoptosis is also found in MCF‐7cells (Figure S4, Supporting Information). Collectively, the excellent CSCs and CC‐targeting effect of the fusion membrane‐coated NPs allowed these nanoeffectors to exert the strong anticancer activity in CSCs and MCF‐7 breast CCs by activating mitochondrial apoptotic pathway and cutting off energy metabolism (ATP).

### 2.4 In Vivo Breast Cancer Progression via Targeting CSCs and Attenuating the Cancer Stemness

**Figure 5:**
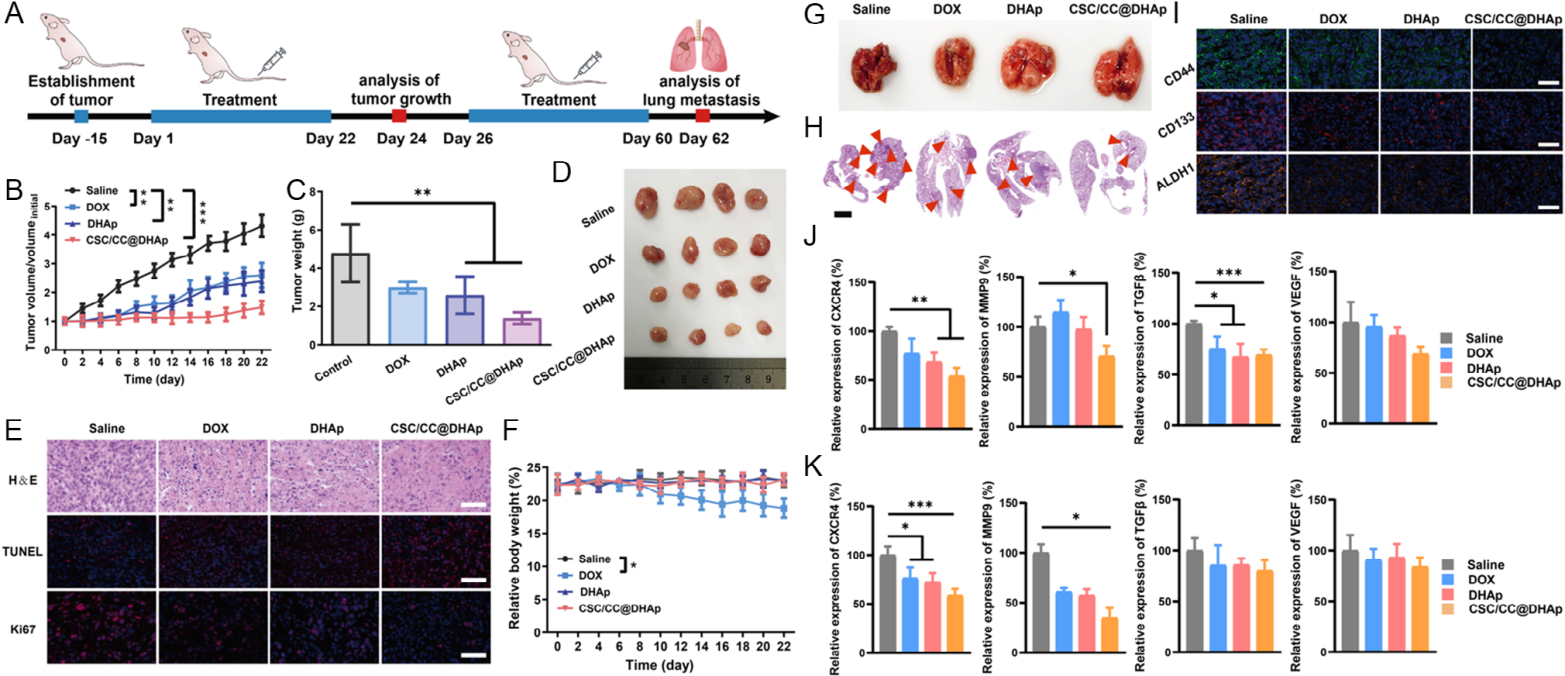
CSC/CC@DHAp nanoeffectors suppressed growth and lung metastasis of primary breast cancer by suppressing the phenotype of tumor stemness. A) In vivo treatment scheme for the subcutaneous breast cancer mouse model (*n* = 4). B) Tumor volume and C) tumor weight curves after the treatment. The tumor volume was measured and normalized to the initial tumor volume on day 1. D) Representative photos of the tumor tissues collected from the nude mice after various treatments. E) H&E, TUNEL, and Ki67 staining images of tumor sections from tumor‐bearing nude mice after different treatment. Scale bar: 50 μm. F) Survival curves for mice (*n* = 6 biologically independent mice per group). G) Representative photographs showing the metastatic tumor nodules in the excised lung tissues. H) Representative H&E staining analysis of the lung tissues, and metastatic nodules were marked with orange arrows; scale bar: 2 mm. I) CD44, CD133, and ALDH1 staining of tumor tissue collected after the indicated treatments. Scale bars: 50 μm. J,K) Expression level of metastasis‐associated proteins including CXCR4, VEGF, TGF‐β, and MMP9 in tumor tissue (J) and lung tissue (K) measured by ELISA assay, *n* = 4. *P < 0.05, **P < 0.01, and ***P < 0.001.

We next evaluated the therapeutic effects of CSC/CC@DHAp NPs in vivo using the tumor xenograft mouse model. MCF‐7 cells were injected subcutaneously into nude mice, and allowed to grow until the tumor volume reached ≈50 mm^3^. Thereafter, tumor‐bearing mice were treated with intravenous injections of saline (as the control), free DOX, DHAp, or CSC/CC@DHAp NPs every 3 days. On day 24, four mice from each group were sacrificed to assess the antitumor efficacy, as illustrated in **Figure** **5**A. As expected, CSC/CC@DHAp NPs exhibit a remarkable therapeutic efficacy. Results show that mice in the control group which received saline treatment suffer from an uncontrollable tumor growth, evidenced by a 4.3‐fold increase in tumor volume compared to its initial volume and a final weight of 4.8 g after 24 days posttreatment (Figure 5B,C). Free DOX and DHAp harvest comparable tumor suppression potency, acquiring 2.6‐ and 2.4‐fold larger final tumor volumes and final weights of 3.0 and 2.6 g, respectively. Notably, CSC/CC@DHAp NPs outperform above three groups, and it almost completely inhibits the growth of MCF‐7 breast cancer tumor, which is ascribed to specific fused membrane‐enabled targeting accumulation of CSC/CC@DHAp NPs in tumors. As indicated in the photos of tumor tissue (Figure 5D), the variation trend of tumor size is consistent with that of either tumor volume or tumor weight, which intuitively demonstrates the antitumor growth effect of CSC/CC@DHAp NPs in vivo.

Furthermore, H&E, TUNEL, and Ki67 staining of tumor sections was used to further explain the therapeutic outcomes. H&E staining images find the empty tissue structure, decreased cell number, and nuclear condensation in tumor treated with CSC/CC@DHAp NPs in comparison with the control group (Figure 5E). TUNEL staining also verifies that CSC/CC@DHAp NPs trigger more tumor apoptosis than other treatments. Ki67 expression is observed to be significantly decreased after the treatment of CSC/CC@DHAp NPs, suggesting the inhibition of tumor cell proliferation. These results suggest that the administration of CSC/CC@DHAp NPs can lead to efficient suppression of tumor growth. Although free DOX can suppress tumor growth to some extent, its systematic administration may pose severe adverse effects, as demonstrated by the remarkable decline in body weight of mice (Figure 5F). In contrast, the administration of CSC/CC@DHAp NPs fails to elicit significant systemic toxicity at the prescribed experimental dose, as evidenced by serum biochemistry assays and histological analyses of major organs (Figure S5, Supporting Information). These results suggest that the biomimetic nanoeffectors have an ideal biocompatibility profile. In addition, when we assessed the in vivo distribution of CSC/CC@DHAp NPs within tumor tissues and major organs as depicted in the above section, CCs–CSCs fusion membrane‐coated NPs exhibit remarkably enhanced accumulation in the tumor and less enrichment in the spleen and lung (Figure 3G,H), suggesting the enhanced efficacy and potentially reduced toxicity in this administration manner.

### 2.5 Attenuation Verification of Cancer Stemness and Plasticity In Vivo

We further assessed whether this therapeutic regimen suppressed the tumor stemness phenotype, a key driver of tumorigenesis to realize tumor progression inhibition. The mice that underwent a 24 day treatment were subsequently subjected to additional treatments to evaluate the progression of lung deterioration, according to the experimental schedule in Figure 5A. During the experimental period, above formulations were intravenously injected via the tail vein at regular intervals (once per 4 days) until day 62. Compared to mice with saline treatment, those mice receiving DOX, DHAp, and CSC/CC@DHAp treatments show evidently reduced lung metastasis, as evidenced by photos including whole lungs tissue and H&E stained lung slices (Figure 5G,H). Importantly, the largest decrease magnitude of lung metastasis is obtained in the CSC/CC@DHAp group, and the average number of metastasis foci in lungs dramatically declines from ∼15 per mouse (saline) to ∼3 per mouse (CSC/CC@DHAp) (Figure S6, Supporting Information).

It has been reported that CD44 and CD133 receptors are overexpressed in CSCs and highly correlated with the degree of cancer stemness.^[^ ^32^ ^]^ Moreover, ALDH1 has been identified as an essential regulator of the growth and differentiation of CSCs.^[^ ^33^ ^]^ Therefore, the expression levels of CD44, CD133, and ALDH1 were monitored to investigate the potential roles of parent CCs–CSCs hybrid membrane‐coated NPs in the regulation of cancer stemness. Results indicate that CSC/CC@DHAp NPs attenuate the cancer stemness, as manifested by the dramatic downregulation in the fluorescence intensity of CD44, CD133, and ALDH1 relative to that in saline treated tumors (Figure 5I). Decreased expression levels of metastasis‐associated proteins including CXCR4, VEGF, TGF‐β, and MMP 9 levels in tumor and lung tissues further demonstrate the reversal of cancer stemness in tumor by such CSC‐targeted nanoeffectors. In detail, compared with saline treatment, CSC/CC@DHAp treatment leads to 44.4%, 81.3%, 71.8%, and 60.3% reduction in the expression of CXCR4, MMP 9, TGF‐β, and VEGF in tumor tissue (Figure 5J), and 65.8%, 45.2%, 83.5%, and 73.7% reduction in lung tissue (Figure 5K). Taking together, such biomimetic CSC‐targeting nanoeffectors display a biological modulation of stemness and plasticity in breast CCs, thus impeding tumor progression, which make them behave as a highly desired therapeutic regimen in preventing tumor recurrence and metastasis.

### 2.6 Early Metastasis Inhibition Evaluation

**Figure 6:**
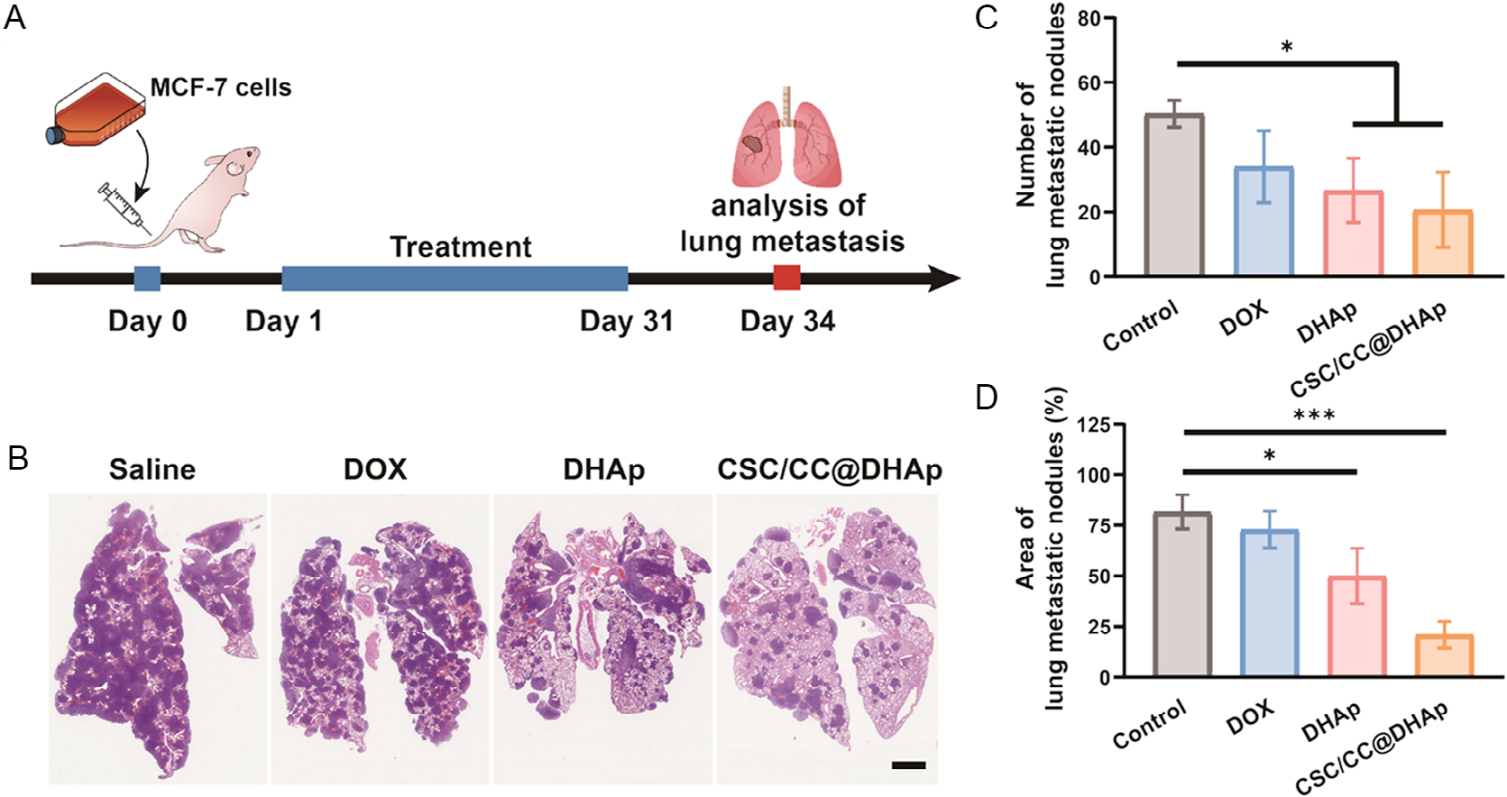
CSC/CC@DHAp nanoeffectors prevented the formation of early metastasis. A) In vivo treatment scheme for metastatic nude mouse model (*n* = 4). B) Representative H&E staining analysis of the lung tissues; scale bar: 2 mm. C,D) Numbers (C) and area (D) of lung metastatic nodules of mice (*n* = 4). *P < 0.05 and ***P < 0.001.

Breast cancer is a malignancy that exhibits high metastatic potential.^[^ ^8^, ^10^, ^34^, ^35^, ^36^, ^37^, ^38^, ^39^, ^40^, ^41^, ^42^, ^43^, ^44^, ^45^ ^]^ As CCs transfer to distant sites, therapeutic interventions become considerably complex and ineffective, and thus, early prevention is of great importance. We then evaluated the impact of biomimetic CSC/CC@DHAp NPs on the formation of early deterioration points. The mice were injected with MCF‐7 cells via the tail vein to imitate the early metastasis process, and starting from the second day, mice are injected with free DOX, DHAp, and CSC/CC@DHAp NPs at 3 day intervals until day 31 (**Figure** **6**A) when their lung tissues were excised for histopathological analysis of metastasis. Lung metastasis was almost completely distributed in PBS and DOX‐treated mice. As a comparison, the number of lung nodules in CSC/CC@DHAp‐treated mice exhibits a considerably decline (Figure 6B). Quantitative data also reflect it, as confirmed by the significantly decrease in numbers and areas of lung metastatic nodules (Figure 6C,D and S7, Supporting Information). This effect might be attributed to their CSC‐targeting capability, which consequently hindered their proliferation and suppressed the CSC‐initiated early malignancy metastasis. These findings demonstrate that the CCs–CSCs fusion membrane‐coated NPs can serve as a potent prophylactic against the early progression of breast cancer.

## 3 Conclusions

In this study, we constructed CCs and CSC‐targeting CSC/CC@DHAp nanoeffectors and accordingly presented a simple and intelligent strategy based on CCs–CSCs fusion membrane coating to targetedly attenuate or reverse cancer stemness and plasticity. Such CCs–CSCs fusion membrane‐coated nanoeffectors exhibited a favorable targeting capability toward breast CCs and CSCs both in vitro and in vivo. By combining with chemotherapeutic drugs, CSC‐inherited fusion membrane‐coated NPs downregulated the expressions of some target proteins associated with cancer stemness and metastasis, activated mitochondrial apoptosis, disrupted energy metabolism in CSCs and CCs, which eventually attenuated or reversed cancer stemness and the induced plasticity, and attained a significant inhibition of tumor proliferation in tumor xenograft mouse model. More importantly, based on the two cancer metastasis models, these biomimetic nanoeffectors hindered breast cancer progression including growth, recurrence, and metastasis by suppressing CSC‐initiated malignancy, which, as we believe, provides an encouraging and general approach to treat malignant tumors by regulating the cancer stemness and plasticity of malignant cancer, holding a high translation potential for repressing malignancy metastasis.

## 4 Experimental Section

All experimental details have been added in Supporting Information.

## Conflict of Interest

The authors declare no conflict of interest.

## Author Contributions

X.D. and K.Z. conceived and designed this project. X.D., Q.Y., H.W., C.Z., T.W., C.F., and Y.Z. performed the experiments. X.D. and K.Z. analyzed and tackled the data. X.D. arranged the figures and wrote original manuscript. K.Z., J.Y., and Q.Z. reorganized and revised the manuscript. K.Z., J.Y., and Q.Z. supported this project. K.Z. supervised and supported the project. All authors commented on this manuscript.

## Supporting information

Supplementary Material

## Data Availability

The data that support the findings of this study are available from the corresponding author upon reasonable request.
