# Supplementary material for: Targetedly Attenuating Cancer Stemness and Plasticity by Homologous Cancer Stem Cell‐Inherited Fusion Membrane Nanoeffectors against Cancer Metastasis
Source: Small Sci. 2023 Dec 3;4(2):2300111. doi: 10.1002/smsc.202300111 (PMC11935028; doi:10.1002/smsc.202300111)
Supplement: Supplementary file 1 — Supplementary Material [file SMSC-4-2300111-s001.pdf]

Targetedly Attenuating Cancer Stemness and Plasticity by Homologous Cancer Stem Cells-Inherited Fusion Membrane Nanoeffectors Against Cancer Metastasis

Xiulin Dong, Qiaoling Yang, Hai Wang, Chunyan Zhu, Taixia Wang, Chao Fang, Yan Zhang, Jianjun Yang, Kun Zhang* and Qing Zhao**

Section A: Materials and methods

Chemicals and biological materials

$Ca(NO_3)_24H_2O$, $(NH_4)_2HPO_4$, ethanol, and Dimethyl sulfoxide (DMSO) were obtained from Shanghai Lingfeng Chemical Reagent Co., Ltd (Shanghai, China). 3-aminopropyltriethoxysilane (AMPTES), NH_2-$(CH_2)_3$-$Si(OC_2H_5)_3$ were purchased from Shanghai Xinming Pharmacy Industry (Shanghai, China). DMEM/F12, B27, bFGF, EGF, insulin and fetal bovine serum (FBS), 4',6-diamidino -2-phenylindole (DAPI) and fluorescein isothiocyanate (FITC) were purchased from Thermo Fisher Scientific (IL, USA). Bovine serum albumin (BSA) were obtained from Sangon Biotech (Shanghai, China). Anti-cleaved caspase 3 antibody and anti-caspase 3 antibody, anti-Bid antibodies, were obtained from Abcam (MA, USA). Rabbit monoclonal anti-β-actin antibodies and horseradish peroxidase (HRP)-conjugated secondary antibody were purchased from Cell Signaling Technology (MA, USA). BCA protein assay kit, Cell Counting Kit-8, Annexin V-FITC/PI assay kit, ATP assay kit were obtained from Beyotime Institute of Biotechnology (Haimen, China).

Cell culture

Human breast cancer cells (MCF-7) and humor liver cells (L-02) were purchased from the Cell Bank of Type Culture Collection of the Chinese Academy of Sciences. Cells were maintained in DMEM supplemented with 10% (v/v) fetal bovine serum under a humidified atmosphere containing 5% CO_2 at 37°C.

Mammospheres of MCF-7 cells were cultured to enrich cancer stem cells (CSCs). Briefly, MCF-7 cells at a concentration of 1000 cells/mL were cultured in suspension with serum-free DMEM/F12, supplemented with 2% B27, 20 ng/mL bFGF, 20 ng/mL EGF, 0.4% lowendotoxin bovine serum albumin (BSA) and 4 mg/mL insulin.

Cancer cell and cancer stem cell membrane extraction and purification

The preparation of the cell membrane was carried out using the method of hypotonic lysing and repeated freeze-thaw with minor modifications as previously reported.[1,2] Briefly, 1×10^7 MCF-7 cancer cells (CCs) or CSCs were resuspended in 1 mL of hypotonic lysing buffer containing EDTA-free protease inhibitor, 10 mM of Tris, and 10 mM of $MgCl_2$ for 3 h on ice. The resulting cell homogenates were subjected to repeated freeze-thaw cycles (frozen in liquid nitrogen and thawed at 37°C in a water bath at least five times) and subsequently centrifuged at 850 g for 10 min at 4°C to remove intracellular precipitates. The supernatant was then isolated and centrifuged at 18,000 g for 60 min. The resulting final precipitate was isolated as the purified CCs and CSCs membranes, and the protein content was quantified using a BCA protein assay kit.

Synthesis and characterization of HAp, DHAp and CSC/CC@DHAp nanoparticles

Hydroxyapatite nanoparticles (HAp NPs) were synthesized using the aqueous precipitation method as previously reported.[3-5] A mixture of 400 mL of $Ca(NO_3)_2$ solution and 240 mL of $(NH_4)_2HPO_4$ solution, with a Ca/P ratio of 1.67, was prepared. $NH_3{\cdot}H_2O$ was subsequently added in a suitable amount to maintain the pH value at 10. The reaction was stirred slightly for 30 h at 10°C. The obtained precipitates were collected by means of centrifugation at 10,000 rpm for 10 min, followed by triple alternating washes with water and ethanol. The precipitates were then subjected to freeze-drying for 24 h. Following this, the synthesized products were calcined at 550°C for 2 h to yield the HAp NPs. The preparation of DHAp nanoparticles was carried out according to the procedure previously reported by us.[5] A total of 10 mg of HAp NPs was introduced into 5 mL of DOX aqueous solution with a concentration of 600 μg/mL. The mixture was stirred continuously in the dark at room temperature for 72 hours. The DOX-loaded nanoparticles were harvested via

centrifugation at 10,000 g for 10 min, subsequently washed with deionized water. After that, the precipitation was freeze-dried to obtain DHAp NPs.

To obtain CSC/CC@DHAp NPs, the extracted MCF-7 cancer cell and cancer stem cell membranes were used to coat DHAp NPs by fusing cell membranes with DHAp NPs suspension and extruding repeatedly via an Avanti miniextruder. The cancer cell membranes and CSC membranes were mixed with equal weight, and sonicated at 100 w for 10 min to acquire fusion cell membranes. Then the aforementioned cell membranes with a concentration of 1 mg/mL, were mixed with NPs at a membrane-to-core weight ratio of 1:1. The mixture was then sonicated on ice at 100 W for 30 seconds, following a sonication cycle of 1 second with intervals of 2 seconds. After that, the mixture was extruded at least 10 times through a 0.8 μm polycarbonate membrane to obtain CSC/CC@DHAp NPs. Meanwhile, the supernatant was collected, and the concentration of free DOX was determined using microplate reader with the detection wavelength of 485 nm according to the standard curve, and the loading efficiency (LE) of DOX in the supernatant was calculated by the formula as follows:

$$\text{LE (\%)}=\frac{\text{initial weight of DOX (μg)-weight of DOX in supernatant (μg)}}{\text{weight of CSC/CC@DHAp (mg)}}\times 100$$

Similarly, the encapsulation efficiency (EE) of DOX in the supernatant was calculated by the formula as follows:

$$\text{EE(\%)}=\frac{\text{initial weight of DOX (mg)-weight of DOX in supernatant (mg)}}{\text{initial weight of DOX (mg)}}\times 100$$

The sodium dodecyl sulfate-polyacrylamide gel electrophoresis (SDS–PAGE) method was used to confirm the existence of membrane- associated proteins on the DHAp NPs. The collection of CSC/CC@DHAp NPs involved a centrifugation step at 10000 rpm, 15 minutes, 4°C to remove the

uncoated cell membranes. The DHAp NPs, CSC@DHAp, CC@DHAp, CSC/CC@DHAp NPs was mixed with SDS loading buffer and heated at 100°C for 5 min. Following quantification using the BCA assay kit, equal amounts of protein (20 μg) from the samples were loaded onto the wells of a 10% SDS-PAGE gel. Subsequently, electrophoresis was performed to separate the proteins based on their molecular weight. The protein bands were then visualized by staining the gel with Coomassie blue dye for a duration of 2 hours. After staining, the gel was destained for 12 h before capturing a photograph.

The size and morphology of the nanoparticles were analyzed through direct observation by transmission electron microscopy (TEM) (JEOL JEM-2100, Tokyo, Japan). A dynamic light scattering system (Malvern Instruments Ltd, Worcestershire, UK) was employed to assess the hydrodynamic size and zeta potential of the surface charge. Fourier transform infrared (FTIR) spectra of the nanoparticles were acquired using a Nicolet 6700 spectrometer (Thermo Fisher Scientific, MA, USA).

In vitro drug release from CSC/CC@DHAp NPs

Various concentrations of DOX aqueous solutions were added to a 96-well plate and analyzed using UV-vis spectroscopy at a detection wavelength of 485 nm. The quantification of DOX concentration was determined by referencing a standard curve. CSC/CC@DHAp NPs were suspended in PBS (3 mL) with varying pH values (5.0 and 7.4) and pleased into a dialysis bag. The dialysis bag containing the NPs was then transferred in 10 mL of PBS solution with the same pH, and placed in an incubator shaker operating at 150 rpm and 37°C. At specific time intervals, 9 mL of dialysate was collected from the system, and an equivalent volume of fresh PBS was added as a replacement. The concentration of DOX in the dialysate was determined using UV-vis spectrophotometry, and the

released DOX concentration was calculated by referencing a standard curve.

Cell viability assay

Cells were plated in 96-well plates at a density of 5×10^3 cells per well and allowed to adhere for 12 hours. Following cell adhesion, the culture medium was removed, and the cells were treated with free DOX, DHAp, and CSC/CC@DHAp NPs at an equivalent DOX concentration for a duration of 24 hours. At the end of incubation, to each well, 20 μL of CCK-8 solution was added, and the cells were subsequently incubated for an additional 2 hours at 37°C, and the absorbance was detected by a microplate reader at 450 nm. The experiments were carried out in four replicates, and cell viability was determined by calculating the percentage of viability in comparison to the control cells.

Annexin V-FITC/PI double staining assay

The Annexin V-FITC/PI assay kit was used to assess the effects of CSC/CC@DHAp NPs on cell apoptosis. Cells were seeded in 6-well plate at the density of 1×10^6 cells per well at 37°C for 12 h, followed by free DOX, DHAp, CSC/CC@DHAp NPs treatment. After 24 h of incubation, cells were rinsed with ice-cold PBS, trypsinized, and collected for staining with Annexin V-FITC and PI following the guidelines provided by the manufacturer. Subsequently, the fluorescence emitted by FITC and PI from a population of 1×10^4 cells was quantified using a flow cytometer (BD Biosciences, CA, USA) within a 30-minute timeframe

Measurement of intracellular ATP level

The intracellular ATP concentration was analyzed using an ATP assay kit, following the instructions provided by the manufacturer. Cells were cultured in 6-well plates and incubated with DHAp, CSC/CC@DHAp NPs, and free DOX (0.31 μg/mL) for 24 h. Following the incubation period, the medium was aspirated, and the cells were washed multiple times with ice-cold PBS. Subsequently,

the cells were treated with the supplied lysis buffer and centrifuged at 10,000 g for 15 minutes at 4°C. The liquid portion (supernatant) was collected for the purpose of quantifying the ATP concentration. Following the reaction, the luminescence was measured using a luminometer (Thermo Fisher Scientific, Varioskan™ LUX, MA, USA), the concentration of ATP was determined by referencing a standard curve. The intracellular ATP level was standardized by normalizing it to the protein concentration in each sample, which was detected by the BCA protein assay kit.

In vitro characterization of cancer targeting

To evaluate the targeting effect of NPs, FITC-labeled CSC/CC@DHAp NPs were prepared. FITC-labeled HAp NPs were first fabricated using a protocol previously reported.[6] In brief, 0.1 g of HAp NPs and 20 mL of AMPTES were combined in 120 mL of anhydrous ethanol and stirred at a temperature of 75°C for a duration of 3 hours. Subsequently, 0.05 g of FITC was introduced and the reaction was allowed to proceed for an additional 6 hours. The obtained powders were filtered and subjected to multiple washes with anhydrous ethanol and de-ionized water. Subsequently, the powders were dried by exposing them to air for a period of 30 minutes, resulting in the formation of dry powders. FITC-labeled HAp NPs were then coated with MCF-7 cancer cell and cancer stem cell membranes as described in section 2.4 in details to obtain FITC-labeled CSC/CC@DHAp NPs.

MCF-7 cells, CSCs, and L-02 cells were seeded in confocal dishes and cultured overnight before treatment with FITC-labeled CSC/CC@DHAp NPs for 4 h. Subsequently, the cells were washed with PBS, stained with DAPI, fixed with glutaraldehyde (0.25%) for 15 min at room temperature, and finally visualized by confocal laser scanning microscopy (CLSM).

Bio-TEM was also utilized to measure the capability of nanoparticles targeting to MCF-7 cells, CSCs and L-02 cells. These cells were exposed to CSC/CC@DHAp NPs at 37°C for 4 h and then

harvested in a glutaraldehyde solution (0.25%) at 4°C. Cells underwent dehydration, embedding, and slicing procedures, and then were subjected to positive staining using uranium salts and lead salts, and finally observed and captured by TEM

MCF-7 cells, CSCs and L-02 cells were seeded in 6-well culture plates, incubated for 12 h, and further incubated with FITC labeled nanoparticles for 4 h. Single cell suspensions were prepared by trypsinizing the cells and subsequently washing them three times with phosphate-buffered saline. The resulting samples were then analyzed using a flow cytometer (BD Biosciences, USA), and the acquired data was analyzed utilizing FlowJo Software (Tree Star, USA). The fluorescence emitted by nanoparticles within cells was quantified to assess the specific targeting of the NPs towards cancer cells.

In vivo characterization of cancer targeting

Tumor-bearing mice were intravenously injected with free DOX, DHAp, and CSC/CC@DHAp NPs. Subsequently, the fluorescence signals of DOX were monitored in all mice at 1, 6, 12, 24, 36, and 48 hours post-injection using a small animal imaging system (Perkin Elmer, MA, USA). Following 48 hours of fluorescence imaging, the mice were euthanized to obtain major organs (heart, liver, spleen, lung, and kidney) as well as the tumor for *ex vivo* fluorescence imaging. Additionally, the major organs and tumor were collected to measure the calcium content using ICP-OES, and blood samples were analyzed for biochemical analyses.

In vivo anti-tumor studies of CSC/CC@DHAp NPs

All animal experiments conducted in this study adhered to the guidelines set forth by the Regional Ethics Committee for Animal Experiments and the approved care regulations of the administrative committee of laboratory animals at Shanghai Tenth People's Hospital (Approval number:

SHDSYY-2022-6962). Female BALB/c nu/nu nude mice, with an average age of 6 weeks and weighing between 18-20 g, were procured and raised at the Laboratory Animal Center, Shanghai Tenth People's Hospital. In order to establish the MCF-7 tumor xenograft model, a suspension containing 1×10^7 MCF-7 cells in 0.1 mL of saline mixed with 0.1 mL of Matrigel (BD Biosciences, CA, USA) was subcutaneously injected into the right mammary fat pads of nude mice. Approximately 15 days following tumor inoculation, when the tumor volume reached approximately 50 mm^3, the mice bearing the tumors were utilized for *in vivo* anti-tumor studies. The tumor-bearing mice were randomly divided into four groups (n = 4 mice) and received intravenous injections via the tail vein once every three days for a total of eight times. The injections included saline, free DOX, DHAp, and CSC/CC@DHAp NPs, all at DOX dose of 5 mg/kg, diluted in 0.2 mL of saline. Every two days, the body weight of the mice and the size of the tumors (L, representing the long diameter; W, representing the short diameter) were measured. The tumor volume (V) was calculated using the formula: $V = L \times W^2/2$. After 24 days, the mice were euthanized, and tumor tissues along with major organs were excised for hematoxylin and eosin (H&E) staining. The apoptotic and proliferative activities in the tumor tissues were evaluated through TUNEL and Ki67 staining, respectively, following the instructions provided by the manufacturer.

Lung metastasis analysis

The postoperative breast tumor-bearing mice, where the treatments were performed as described above, were further intravenously injected via the tail vein once in 4 days, with saline, free DOX, DHAp and CSC/CC@DHAp NPs, and all mice were sacrificed on day 62, the lungs were isolated, photographed, and then subjected to H&E staining, the surface nodules of lung metastasis were counted. Subsequently, the lungs and tumor were harvested for enzyme linked immunosorbent assay

(ELISA). The concentrations of TGF-β, MMP 2, VEGF and CXCR4 in the culture supernatant of each tumor and lung were measured following the instructions provided by the manufacturer (Cusabio, Wuhan, China). Following the completion of the reaction, a measurement was taken at a wavelength of 450 nm using a microplate reader. The concentration of the analyte was then determined by referencing a standard curve. Simultaneously, immunohistochemical staining was performed on tumor sections to analyze the expression of CSC markers, including CD44, CD133, and ALDH1. The stained tissues were subsequently visualized and imaged using a CLSM.

Metastasis prevention assay

Female nude mice, with an average age of six weeks, were injected with 1×10^6 MCF-7 cells through the tail vein (n = 4). After that, saline, free DOX, DHAp and CSC/CC@DHAp NPs at the DOX concentration of 5 mg/kg were intravenously injected at three-day intervals one day after MCF-7 cells injection. 34 days after treatment, the lungs were excised for H&E staining as described in above section.

Statistical analysis

All data were presented as mean ± standard deviation (SD) and derived from a minimum of three independent experiments. Student's t-test was performed to compare differences between two groups, while one-way analysis of variance (ANOVA) was used for multiple comparisons. Significance levels were denoted as *$p < 0.05$, **$p < 0.01$, and ***$p < 0.001$. The Kruskal-Wallis test was employed to compare tumor volumes among different groups.

References

1 Xiao, T. *et al.* Macrophage membrane-camouflaged responsive polymer nanogels enable magnetic resonance imaging-guided chemotherapy/chemodynamic therapy of orthotopic glioma. *ACS Nano* **15**, 20377-20390 (2021).

2 Ning, S. *et al.* A type I AIE photosensitiser-loaded biomimetic nanosystem allowing precise

depletion of cancer stem cells and prevention of cancer recurrence after radiotherapy. *Biomaterials*, 122034 (2023).

3 Sun, Y. *et al.* Mitochondria-targeted hydroxyapatite nanoparticles for selective growth inhibition of lung cancer *in vitro* and *in vivo*. *ACS Appl. Mater. Inter.* **8**, 25680-25690 (2016).

4 Yuan, Y., Liu, C. S., Qian, J. C., Wang, J. & Zhang, Y. Size-mediated cytotoxicity and apoptosis of hydroxyapatite nanoparticles in human hepatoma HepG2 cells. *Biomaterials* **31**, 730-740 (2010).

5 Dong, X. L. *et al.* Synergistic combination of bioactive hydroxyapatite nanoparticles and the chemotherapeutic doxorubicin to overcome tumor multidrug resistance. *Small* **17**, 2007672 (2021).

6 Yuan, Y., Liu, C., Qian, J., Wang, J. & Zhang, Y. Size-mediated cytotoxicity and apoptosis of hydroxyapatite nanoparticles in human hepatoma HepG2 cells. *Biomaterials* **31**, 730-740 (2010).

Part B: Supplementary figures

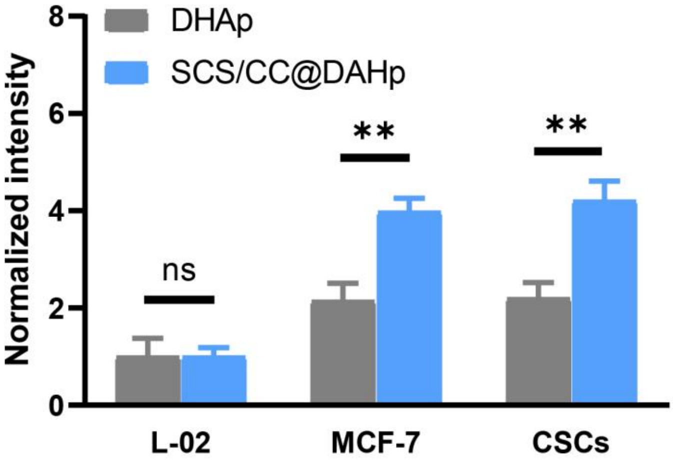

Figure S1 Normalized fluorescence intensity of FITC after incubation with DHAp, CSC/CC@DHAp for 4 h.

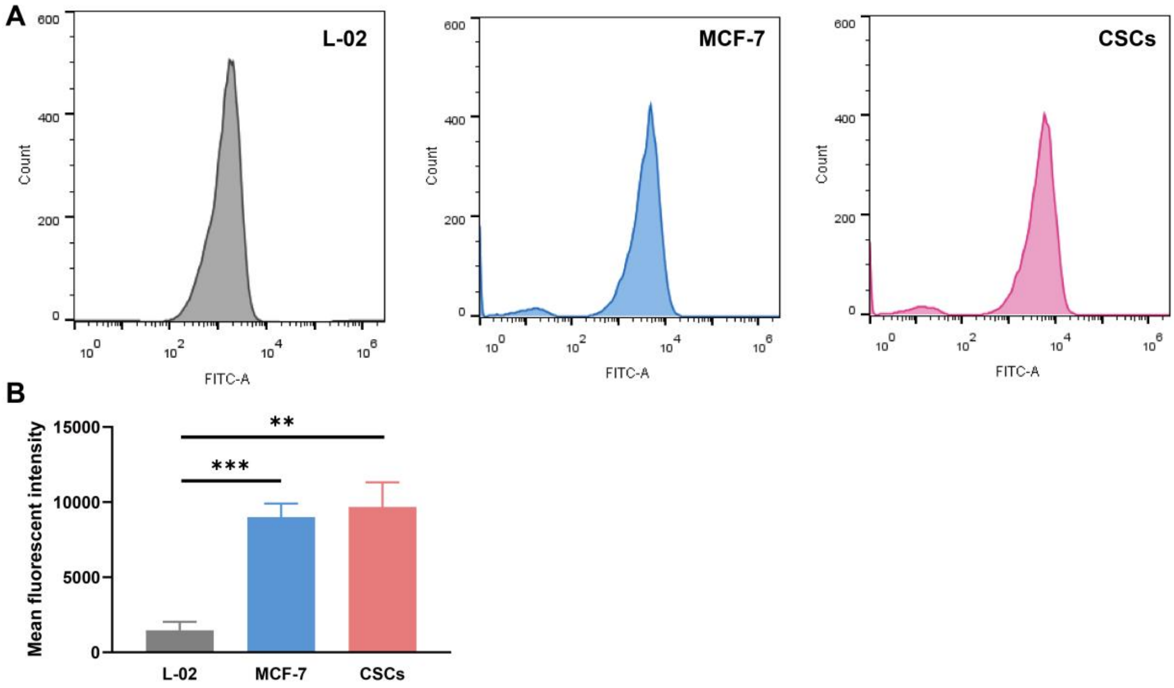

Figure S2 Flow cytometry image (A) and quantification of the mean fluorescence intensities (B) of the histograms in Figure 3B. Error bars showed the standard deviation (n = 3), *P <0.05, **P < 0.01, ***P < 0.001.

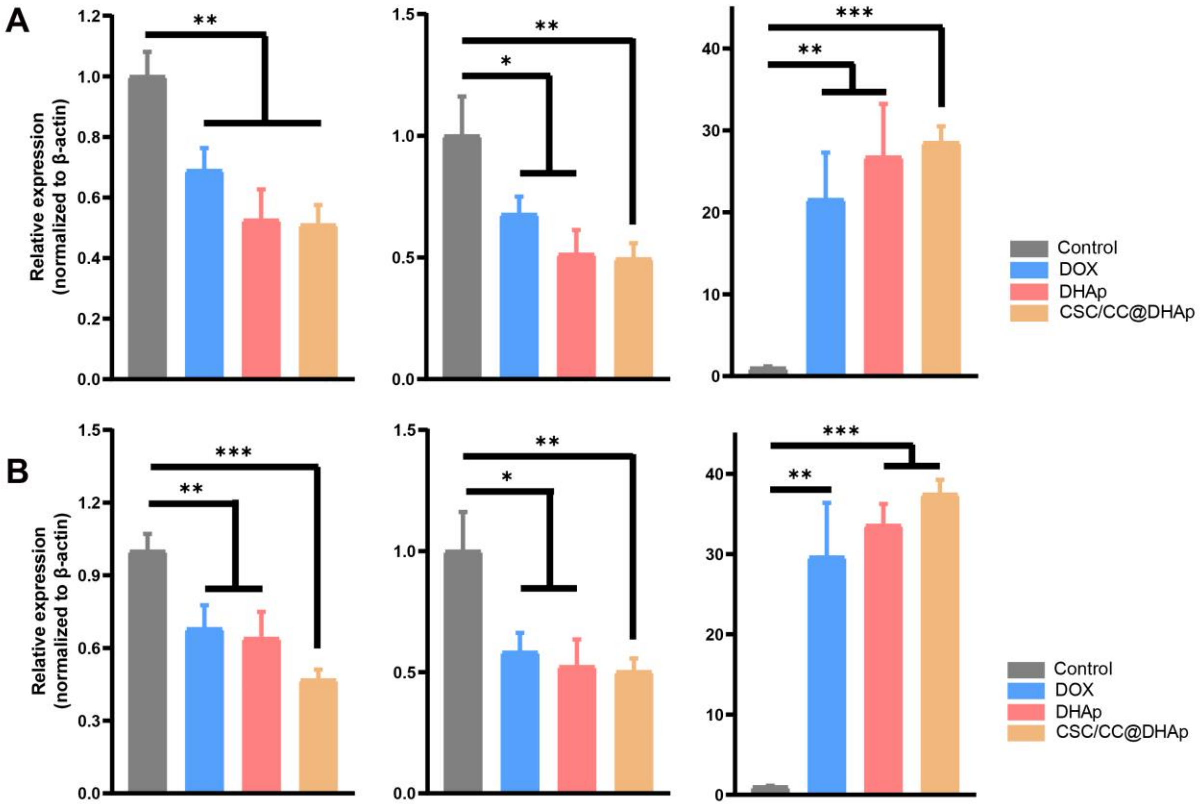

Figure S3 Relative Bid, caspase 3, cleaved caspase 3 expression level in CSCs (A) and CCs (B) quantified from western blot image in Figure 4F, and that were normalized to β-actin. Error bars showed the standard deviation (n = 3), *p < 0.05, **p < 0.01, ***p < 0.001.

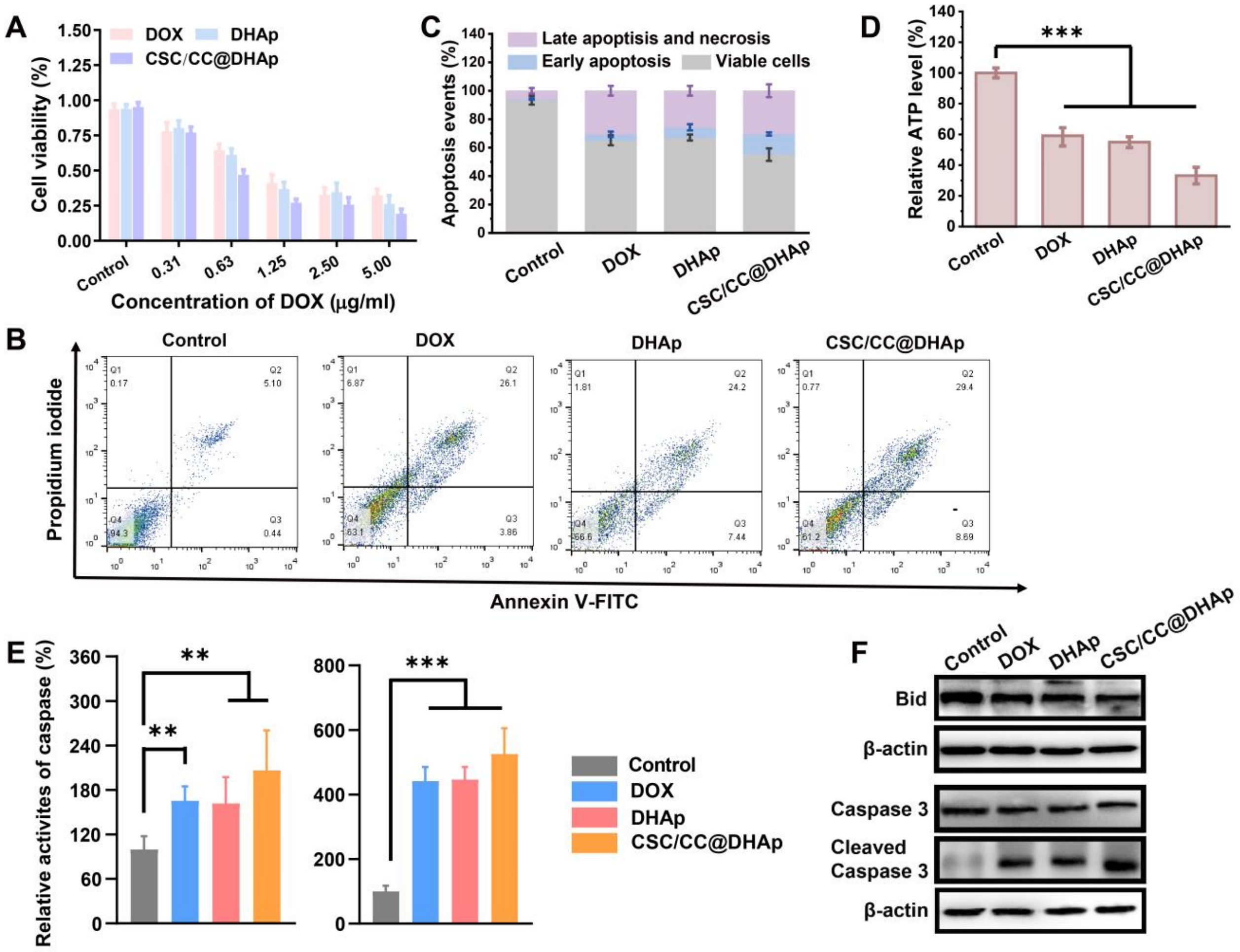

Figure S4 Inhibition of biological activity of breast cancer cells by CSC/CC@DHAp nanoeffector. (A) Cell viability of MCF-7 cells following exposure to free DOX, DHAp and CSC/CC@DHAp NPs, respectively, at various concentrations for 24 h (n = 4). (B) Cell apoptosis analysis of MCF-7 cells detected with flow cytometry. (C) Corresponding apoptosis ratios quantified from figure (B). (D) Intracellular ATP level of MCF-7 cells after incubation with free DOX, DHAp and CSC/CC@DHAp NPs for 24 h. (E) Activity of caspase-3 (left) and caspase -9 (right) in MCF-7 cells (n = 3). (F) Western blot assays of protein caspase3, cleaved caspase 3, and Bcl-2 in MCF-7 cells after different treatments for 24 h.

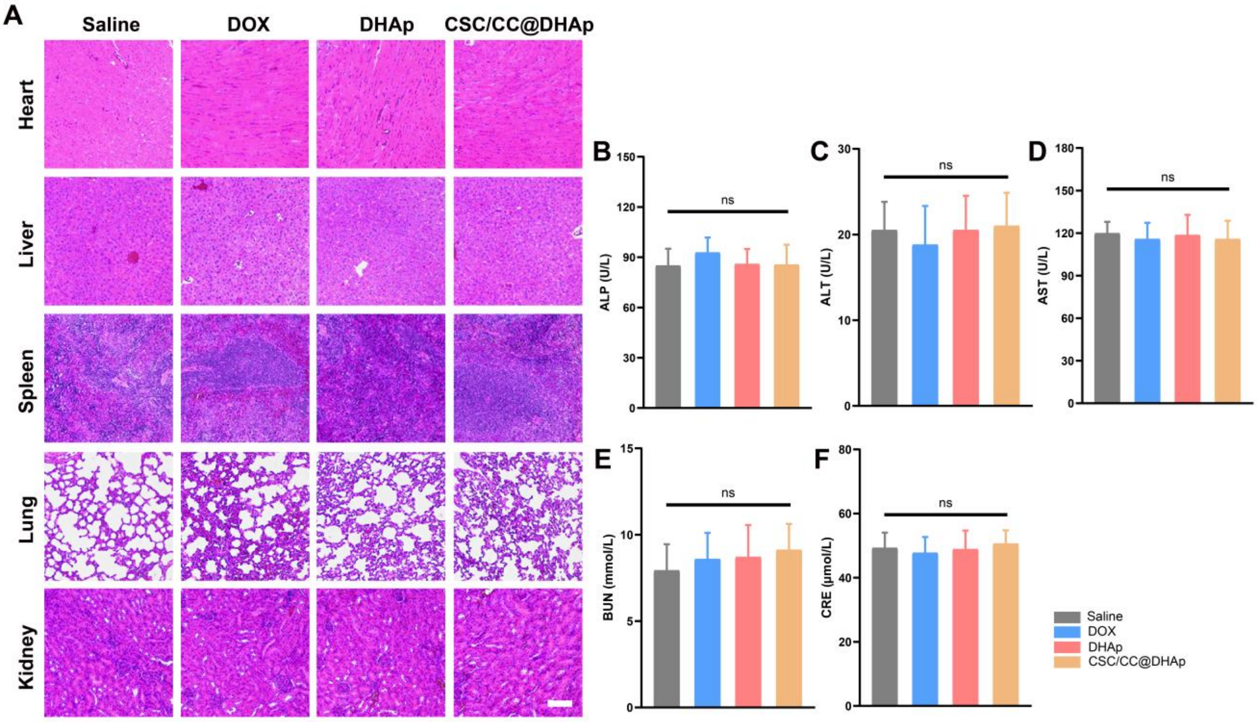

Figure S5 (A) H&E staining images of tissue sections of heart, liver, spleen, lung, kidney in breast tumor-bearing nude mice treated with saline, free DOX, DHAp and CSC/CC@DHAp NPs respectively. (B-F) Blood biochemistry data, including alkaline phosphatase (ALP), alanine aminotransferase (ALT), aspartate aminotransferase (AST), blood urea nitrogen (BUN), creatinine (CRE). Data were presented as means ± SD (n = 3).

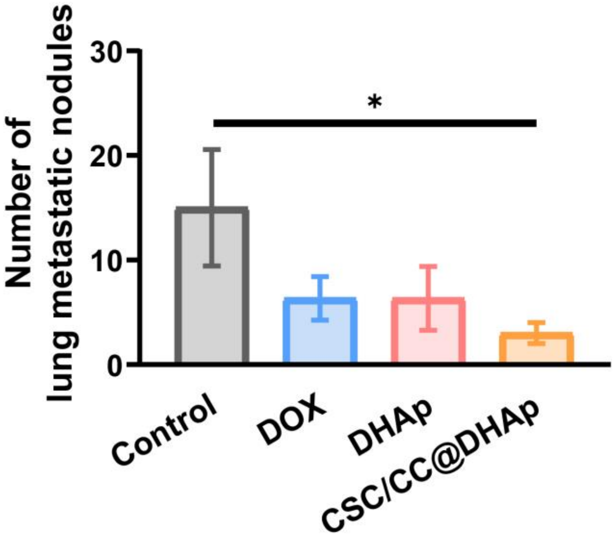

Figure S6 Numbers of lung metastatic foci of mice (n = 4 biologically independent mice per group).

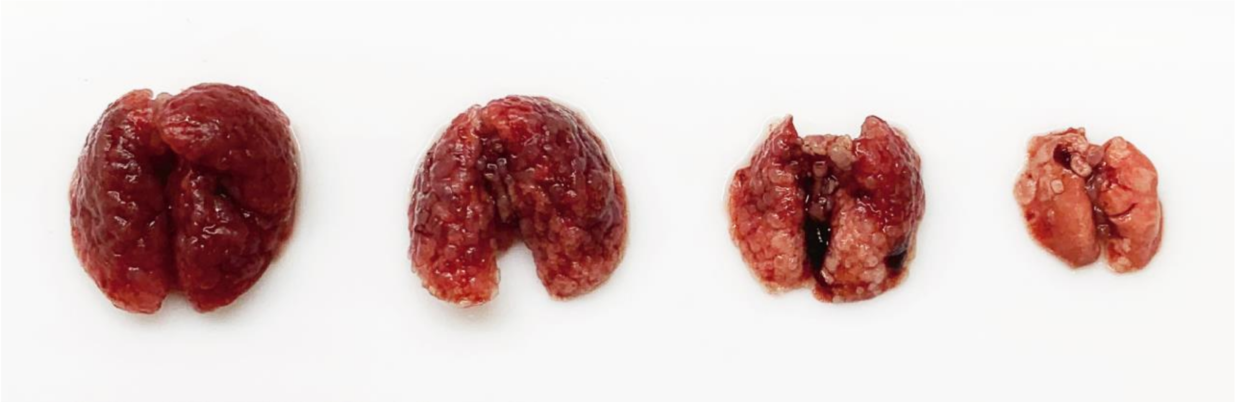

Figure S7 Representative images depicting metastatic tumor nodules in excised lung tissues on day 34.
